# Henipavirus Immune Evasion and Pathogenesis Mechanisms: Lessons Learnt from Natural Infection and Animal Models

**DOI:** 10.3390/v14050936

**Published:** 2022-04-29

**Authors:** Philip Lawrence, Beatriz Escudero-Pérez

**Affiliations:** 1 Science and Humanities Confluence Research Centre (EA 1598), Catholic University of Lyon (UCLy), 69002 Lyon, France; 2 WHO Collaborating Centre for Arbovirus and Haemorrhagic Fever Reference and Research, Bernhard Nocht Institute for Tropical Medicine, 20359 Hamburg, Germany; 3 German Centre for Infection Research (DZIF), Partner Site Hamburg-Luebeck-Borstel, 38124 Braunschweig, Germany

**Keywords:** Nipah virus, Hendra virus, henipavirus, emergence, zoonosis, bat-borne, immune evasion mechanisms, pathogenesis, animal models

## Abstract

Nipah henipavirus (NiV) and Hendra henipavirus (HeV) are zoonotic emerging paramyxoviruses causing severe disease outbreaks in humans and livestock, mostly in Australia, India, Malaysia, Singapore and Bangladesh. Both are bat-borne viruses and in humans, their mortality rates can reach 60% in the case of HeV and 92% for NiV, thus being two of the deadliest viruses known for humans. Several factors, including a large cellular tropism and a wide zoonotic potential, con-tribute to their high pathogenicity. This review provides an overview of HeV and NiV pathogenicity mechanisms and provides a summary of their interactions with the immune systems of their different host species, including their natural hosts bats, spillover-hosts pigs, horses, and humans, as well as in experimental animal models. A better understanding of the interactions between henipaviruses and their hosts could facilitate the development of new therapeutic strategies and vaccine measures against these re-emerging viruses.

## 1. Generalities

The viral species *Nipah henipavirus* (NiV) and *Hendra henipavirus* (HeV) are negative single-stranded RNA viruses that belong to *Paramyxoviridae* family, *Paramyxovirinae* subfamily and, together with *Cedar henipavirus* (CedV), *Mojiang henipavirus* and *Ghanaian bat henipavirus*, form the genus *Henipavirus* (https://ictv.global/taxonomy/ (accessed on 25 January 2022)). Both NiV and HeV are highly pathogenic and can infect a wide range of wild and domestic animals, as well as humans, in whom they can cause a pulmonary or encephalitic henipavirus-mediated disease with observed fatality rates of up to 60% and 92% for HeV and NiV, respectively [1,2].

### 1.1. Genomic Features

**Figure 1:**
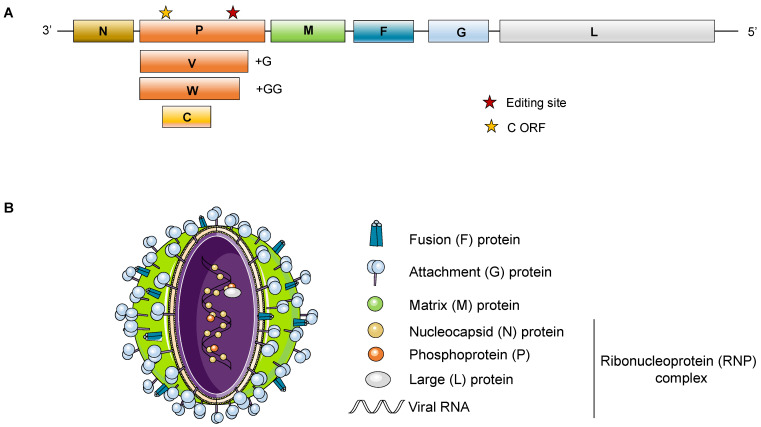
Henipavirus structure and genome organization. (**A**) Schematic representation of henipaviruses single-stranded negative-sense RNA genome containing six genes encoding the nucleoprotein (N), the phosphoprotein (P), matrix protein (M), the fusion protein (F), the attachment glycoprotein (G), and the polymerase protein (L). Additionally, the henipavirus *P* gene encodes three nonstructural proteins: two (V and W) are generated by the addition of one or two G nucleotides in the editing site of the *P* gene, and the C protein is encoded by an alternative open reading frame (ORF); (**B**) Schematic representation of an henipavirus particle. The described structural proteins form the pleomorphic particle. The ribonucleic (RNP) complex is formed by N, P, L proteins and the viral RNA.

Both NiV and HeV have a non-segmented single-stranded negative-sense RNA of around 18.2 Kb that, as shown in Figure 1, codes for 6 structural proteins: the nucleocapsid (N), the phosphoprotein (P), the matrix protein (M), the surface glycoprotein (G) and fusion (F) protein, and finally the viral polymerase (L). Additionally, the *P* gene encodes three non-structural proteins (C, V, W) that are expressed in infected cells [3].

Some of the genomic features that differentiate henipaviruses from the rest of the *Paramyxoviridae* include the length of the viral genome: 18,246 nucleotides (nt) for NiV and 18,234 nt for HeV, which is approximatively 15% longer (around 2700 bases) than other paramyxovirus genomes. Only the rodent paramyxovirus J-virus has a 700 nt longer genome [4]. Henipaviruses are also unique in having extra nucleotides in untranslated regions of the 3’ end of all of their transcription units except for the *L* gene (five out of six) [5,6]. This may explain why all proteins except for the phosphoprotein (P), which is 100–200 amino acids longer than in other members of the family (Respiroviruses and Morbilliviruses) [7,8], are roughly the same size as their counterparts when translated. Despite their difference in length, henipavirus genomes follow the rule of 6 and encode for 6–10 proteins, some of which are derived from overlapping open reading frames (ORFs). Another characteristic of henipaviruses, with the exception of some putative African henipaviruses [9], is that they contain a highly conserved GDNE catalytic site in their polymerase rather than the GDNQ sequence present in the majority of all other negative-strand RNA viruses [5,10]. 

### 1.2. Biological Features

Amongst paramyxoviruses, henipaviruses is the only genus that causes a highly pathogenic disease of zoonotic origin. Other paramyxovirus, such as Menangle virus and Tioman virus are of zoonotic origin, however their pathogenicity has not been shown to be as high as for henipaviruses [11]. Compared to other paramyxoviruses, henipaviruses have a very broad range of hosts including eight species of five mammalian orders (Chiroptera, Artiodactyla, Carnivora, Peressodactyla, Primates): several Pteropid bat species [12,13], pigs [14,15], goats [14,16], cattle [16], cats [14,17], dogs [14], horses [18], and humans [19,20]. In addition to these naturally infected hosts, rodents such as golden hamsters, type I interferon receptor knockout (IFNAR) mice, guinea pigs, ferrets, and non-human primates have also been shown to be experimentally susceptible to NiV infection [21,22,23,24] (see Section 4). Some of these models, such as ferrets and hamsters, are infectable with henipaviruses and reproduce some of the features observed during infection in humans. However, other models, such as mice, are resistant to NiV infection if they are not genetically adapted, as for IFNAR mice, despite the fact that they express cellular receptors susceptible to NiV entry, suggesting that resistance to NiV infection may occur at a post-entry step [25,26,27].

The bridging role of domesticated animals in NiV and HeV transmission between wildlife and people significantly increases the risk of spill-over to humans [28]. As well as the increased risk in viral transmission, the broad range of species affected by henipaviruses and their stuttering chains of transmission raise concerns for public health, as well as economical and animal health costs [29,30].

### 1.3. Bats: Natural Reservoir of Henipaviruses

Bats within the order Chiroptera comprise a large collection of species, classified in two suborders: Microchiroptera (that means small hand-wing), with 18 families, and Megachiroptera (big hand-wing) with only one family, *Pteropodidae*, also known as Old World fruit bats. Whilst bats have been found to host viruses that are not currently known to cause disease in humans, including some hepadnaviruses, morbilliviruses, pegiviruses, and hepaciviruses [9,31,32,33], many other bat viruses have known potential to cause severe disease in humans. Prominent examples are filoviruses, beta-coronaviruses, and henipaviruses that can cause hemorrhagic, encephalitic, or respiratory diseases with high case-fatality rates. There are more than 1300 species of bats [34]. Some bat species are known to be carriers of specific pathogens. For instance, coronaviruses are common in *Hipposideridae* bats [35], although *Severe acute respiratory syndrome-related coronavirus* (SARS) is found in Rhinolophus bats [36,37]; hantaviruses in *Nycteridae* bats [38]; and paramyxoviruses, including henipaviruses [39,40] and filoviruses [41] in Pteropus and Rousettus bats, respectively.

The known distribution of henipaviruses expands globally and overlaps with some of the most populated areas in the world; both henipavirus-like genetic material and antibodies have been detected in many countries (Bangladesh, Singapore, Malaysia, India, Indonesia, Australia, Cambodia, Thailand, Indonesia, Papua New Guinea, China, Madagascar, Ghana, and throughout South America) in different species. There is growing evidence indicating that henipaviruses, despite having mainly been detected in the Pteropus genus, may infect other genera of pteropodid bats and even microbats including *P. lylei*, *P. hypomelanus*, *P. vampyrus*, *P. rufus*, *P. poliocephalus*, *P. scapulatus*, *P. conspicillatus*, *Eidolon dupreanum*, *Eidolon helvum*, *Rousettus madagascariensis*, *Carollia perspicillata,* and in the insectivorous *Hipposideros larvatus* and *Pteronotus parnellii* [9,42,43]. In addition to bats, several reports have suggested that some rodent species may also harbor henipa-like viruses [44,45].

Pteropus is the largest family of the Old World fruit bats or ‘flying foxes’, containing more than fifty species, and it is well known that, amongst other viruses, it is the natural reservoir of both HeV and NiV [46,47]. When HeV was first identified in 1994 in Australia during an outbreak of acute respiratory disease in horses, the reservoir was still unknown [48,49]. It was first believed that the disease was caused by exposure to contaminated biological products or consumption of performance-enhancing substances but these hypotheses were soon discarded, thus supporting the possibility that a wildlife reservoir was responsible for the outbreaks [50,51]. Three humans out of the seven that were in close contact with infected horses and contracted HeV have died between 1994 and 2009. Many terrestrial species were investigated due to the outbreak but they were all discarded in favor of animal species that: (i) had been in the outbreak locations, (ii) had the ability to move between the different outbreak locations, and (iii) could have been in contact with the infected horses. Only several bird species and flying foxes accomplished these criteria, however, due to previous reports of paramyxoviruses harbored in bats (such as parainfluenza type 2 virus, Mapuera virus, and Menangle virus) and the fact that HeV seemed to have a predilection for mammals, flying foxes were prioritized [52,53,54,55]. Soon, *P. alecto*, and shortly after, the other three endemic species of flying foxes in Australia (*P. poliocephalus*, *P. scapulatus*, and *P. conspicillatus*) were identified as natural hosts of HeV [56]. 

In the case of NiV, the virus was first discovered in Malaysia during an outbreak in pigs and humans in 1999 [57]. Since NiV was shown to be molecularly close to HeV, Malaysian bats species were prioritized for surveillance. After screening of blood and tissues from 14 species of Old World fruit bats in Malaysia, *P. vampyrus* and *P. hypomelanus* were identified as the natural hosts of NiV due to the high percentage of individuals found to be seropositive for the virus [12]. This was later confirmed by the isolation of NiV from *P. hypomelanus* in 2002 [39]. From 2001–2005, five outbreaks of NiV-associated disease in humans took place in Bangladesh [58]. They were very similar to the outbreaks observed in Malaysia with the notable difference, however, that the Bangladesh outbreaks were not only not associated with disease in pigs but that clear cases of human-to-human transmission of the virus were observed [58]. In addition, the main clinical difference between NiV infections in Malaysia and Bangladesh was that NiV cases in Bangladesh had on average a shorter and more narrow incubation period than NiV cases in Malaysia; moreover, there were differences in the respiratory involvement during the infection: in Malaysia, 14-29% of patients showed respiratory involvement while in Bangladesh and India, it was seen in 60–75% of the patients. In fact, acute respiratory distress syndrome (ARDS) characterized by a cough and difficulty breathing was mainly described during NiV infections in Bangladesh but not in Malaysia. NiV-infected patients in Malaysia also presented more myoclonus (involuntary muscle spasms) compared to NiV infected patients in Bangladesh [59]. Molecular data supported differences between the Bangladesh outbreak of NiV of 2004 and the Malaysian isolates of 1999: they only shared 92% of identity [60]. Sequences obtained from Malaysian human cases suggested only one source of human infection from an amplifying porcine host whereas the Bangladesh cases have shown multiple introductions of the virus to humans [61,62,63]. Some of the most recent NiV outbreaks have taken place in India in Kerala between 2018 and 2021, one of which exceeded a 90% mortality rate [64,65]. Outbreaks in India have been shown to be caused by NiV strains close to those circulating in Bangladesh [65,66]. Altogether, these data have led to the classification of two strains: Malaysian Nipah virus and Bangladesh Nipah virus.

Henipaviruses can be shed orally, urogenitally, in feces, and in birthing fluids of pteropid bats [67,68]. This implies that mutual grooming, mating, fighting, exposure to feces, urine, or other fluids including saliva, and ingestion of contaminated food are mechanisms of henipavirus transmission both amongst bats and in spillover events to other species [69]. NiV and HeV transmission dynamics have been shown to be potentially associated to birth pulses and influxes of seronegative juveniles in the population leading occasionally to a seroprevalence below the herd-immunity threshold [70,71]. The fact that NiV *N*-gene sequences in bat populations are extremely similar over time compared to those found in humans suggests that henipaviruses may be prevalent and stable in certain bat colonies [72]. 

Spillover events where NiV and HeV viruses are capable of crossing the species barrier to cause disease in humans or other animal species seem to depend on multiple factors that increase the exposure of humans to the pathogen (interspecies transmission), enhance infection in the host, and/or expand transmission amongst individuals of the newly targeted host species (intraspecies transmission). Such “jumps” of viruses between species is often due to either changes in the pathogen, the host, or the environment. In the case of HeV and NiV, similarities in viral RNA sequences isolated from bats, from “bridge” species such as pigs or horses, and from humans would suggest that the largest changes influencing spillover events occur in the environment and involve increased contacts between bats and livestock/humans [73]. Among the more than 200 viruses associated with bats, there are more spillover events observed with RNA viruses; this is probably due to the high mutation rate of these viruses which translates into a higher genetic variability which may aid a fast adaptation to changing environmental conditions in a new host species [74]. Anthropogenic activities have increased human–bat–livestock interactions due to human exploitation of natural habitats [75,76], thereby heightening the emergence and re-emergence of high consequence infectious diseases. In recent years, changing landscapes and deforestation has deeply affected bat roosting sites, thus forcing colonies to change their ecology and behavior and to look for niche expansion, often closer to human locations where foraging or agriculture takes place. There are many circumstances by which humans can be exposed to bats including activities in caves, hunting, or exposure to bats roosting in houses. An example of the latter is the recent discovery of *Bombali ebolavirus* in *Mops condylurus* bats which are frequently found in houses in Guinea [77,78].

## 2. Pathogenesis

### 2.1. Transmission

In recent years, the emergence of new zoonotic viruses with potential to cause disease in humans has increased. Understanding the origin, the causes (including the frequency and nature of interactions between humans and reservoir species such as bats), and the transmission of these pathogens will be crucial in preventing and controlling future emerging infectious diseases. In the case of henipaviruses, it is quite clear how these viruses reach humans. There are mainly three pathways of transmission of HeV and NiV from bats to humans: 

(i) Foodborne transmission: Ingestion of raw contaminated date sap is one of the most common transmission routes for NiV [79]. Cases of transmission mostly take place from December to March, when the palm sap is harvested. During this process, the collection recipients are left exposed to the environment overnight, and many bats take the chance to access and lick the sweet sap [19,80]. Since NiV has been shown to survive in sugar-rich solutions [81], it is believed that the virus is highly stable in the sap. In fact, during the 2005 Nipah outbreak in Bangladesh, 91% of the NiV index patients developed their symptoms during the date palm sap collection season [82,83]. From 2010 to 2014, NiV infections detected in Bangladesh during hospital-based encephalitis screening were shown to be associated with consumption of liquor made with date palm sap [84]. In a study conducted in Bangladesh villages where NiV infections were reported from 2011 to 2013, it was observed that amongst the different drivers for risk of infection considered (including number of palm sap trees, number of bats, humans, and consumption of palm sap), only the consumption of palm sap tree showed a significant correlation with NiV infections [85]. Despite the fact that HeV hosts are also bats, no direct HeV infections in humans due to palm sap consumption have been reported. 

While henipaviruses foodborne zoonoses through consumption of bats or infected animal products from spill-over host species (pigs, horses) are not the most common route of virus transmission, it can pose a threat to human health, either through cross contamination or through the consumption of the infected edible product [86]. 

(ii) Contact with domestic animals: The fact that domestic animals are susceptible to zoonotic viruses thus having the potential for disease transmission to humans is crucial in creating spill-over risks for highly pathogenic viruses. Transmission of NiV to domestic animals takes place when these feed from palm sap or partially eaten fruit contaminated with NiV-containing feces, urine, or saliva. If infected, these domestic animals can then shed the virus and transmit it to humans. The combination of fruit trees, pigs, bats, and humans in the same surroundings facilitates the emergence of henipaviruses. With this model, transmission of henipaviruses to humans requires close proximity. Several further NiV outbreaks in 2001, 2003, and 2004 have been associated with contact with domestic animals [58,80]. In Malaysia and Singapore, transmission has mainly occurred through contact of humans with pigs. However, in the case of NiV outbreaks in Bangladesh (and India), transmission is mainly food-borne originated, mostly due to the consumption of contaminated palm sap [83,87] or due to human-to-human transmission (see Section 3) [88]. In the case of HeV, it appears that only horses become directly infected by bats and thus act as amplifying hosts. It has previously been shown that HeV can be excreted from infected horses up to 72 h before the animals present clinical signs [89]. However, HeV infectivity is low and humans are generally infected only when highly exposed to HeV-infected horse secretions or organs. Despite the fact that bat handlers are frequently exposed to potential pathogens from sick bats by scratches, bites, saliva, feces, etc., no direct transmission of HeV from bats to humans has been reported.

(iii) Human-to-human transmission: there have been multiple NiV outbreaks where human-to-human transmission took place. An example of this occurred in 2004 in Bangladesh when four contacts of a NiV index case developed symptoms 15–27 days after the index case developed the illness and the chain of transmission affected 34 people in total [90]. From 2001 to 2007, it was shown that out of 122 detected NiV cases, 9 patients infected with NiV were responsible for transmitting NiV to 62 other people, meaning that 7% of the patients were directly responsible for infecting 50% of the patients of the outbreak through human-to-human contact [82]. Close physical contact is required for the hands-on care of the patient and generally, due to social norms in NiV endemic regions, it is provided by the family of the patients [91]. Taking into account the risk that human travel may represent, this route of contact should not be overlooked. In addition to physical contact, respiratory secretions are also important for person-to-person NiV transmission. It has been shown that patients coughing are more likely to transmit NiV and that care providers sharing rooms, food, or contact with NiV-infected patients are at a very high risk of contracting Nipah virus infection, especially when there is exposure of carers to the saliva of sick patients [90]. In agreement, epidemiological studies have shown that despite the risk of transmission of NiV through aerosols, close contact is generally necessary for human-to-human transmission [90,92]. These data were confirmed in experimentally NiV-infected hamsters that did not transmit NiV through aerosols [93]. It has also been shown that NiV deceased patients can transmit the virus to people that were in close contact with the body [92]. Since secondary transmitters of NiV infection have not been shown to be better transmitters of the virus when compared to NiV index cases, it is believed that there is no selection of strains predisposed to a better human-to-human transmission and that the different NiV strains have no significant differences in the efficiency of human-to-human transmission but that differences observed in the Malaysia vs. Bangladesh outbreaks are mostly due to different circumstances of transmission. In the case of HeV, no human-to human transmission has been detected to date [94].

**Figure 2:**
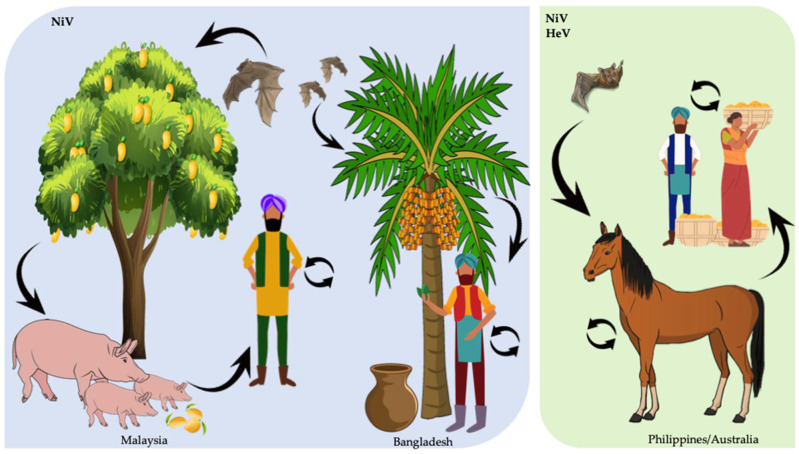
Major routes of NiV and HeV transmission. NiV and HeV natural reservoir are Pteropid spp. bats. NiV transmission in Malaysia mostly occurs when pigs consume partially eaten fruit contaminated with NiV-containing feces, urine, or saliva. Subsequently, humans in close contact with pigs contract NiV and can transmit it to other humans. In Bangladesh, NiV is believed to be transmitted mainly through consumption of date sap. When bats drink from the palm sap stream or collection recipients, they contaminate it with NiV via saliva or urine and humans can contract NiV after consuming contaminated palm sap. Infected people can in turn transmit NiV to other people. In the Philippines and Australia, bats can transmit NiV or HeV to horses which will become amplifying hosts and can transmit the viruses to humans and other horses through close contact.

The most common HeV and NiV transmission pathways are summarized in Figure 2.

Globalization and anthropogenic changes certainly have an influence on the geographical distribution of species with the potential to carry emerging zoonotic pathogens and their interactions with humans. In addition to the transmission pathways mentioned above, there are other modes of transmission such as importation of guano with potential to contain henipaviruses. While it is not the most likely henipavirus transmission situation, the potential risk of transmission to humans should not be neglected [86,95,96,97]. Altogether, the many possible mechanisms of spillover and transmission of henipavirus raise concerns on how to prevent and predict such risk situations. Better surveillance and better knowledge of virus prevalence in infected products or animals and humans would provide a better understanding on the transmission risks associated with henipaviruses.

### 2.2. Pathogenesis of Henipaviruses

Henipaviruses have two glycoproteins: the G protein, which is required for cell attachment, and the F protein that mediates the fusion of the viral membrane with the host cellular membrane. HeV and NiV G glycoprotein is a type II transmembrane protein of 602 and 604 amino acids, respectively, that is very similar to other paramyxoviruses glycoproteins [98,99]. However, contrary to most other paramyxoviruses, henipavirus glycoprotein does not have hemagglutination or neurominidase activities [100] and contrary to respiroviruses, rubulaviruses, or avulaviruses, does not use glycan-based receptors, but instead, like the morbilliviruses, uses protein-based receptors [101]. 

It has been shown that henipavirus glycoproteins, similar to those of other paramyxoviruses, form dimers that can then organize to form tetramers [25,102]. After binding its cellular receptor ephrin-B2 or -B3 for NiV and ephrin-B2 for HeV, changes in the conformation of the protein trigger fusion (by F protein) between cellular and viral membranes [103,104]. Of interest, in the case of CedV, while preserving recognition of ephrin-B2, this virus can uniquely among known henipaviruses also use not only ephrin-B1 for cellular entry [105] but also use ephrin-A2, ephrin-A5, and in some species, ephrin-A1 [106].

Cellular susceptibility to NiV and HeV in vitro depends on expression of ephrin-B2/B3. For instance, NiV can infect porcine microvascular endothelial cells (PBMECs) and human brain endothelial cells (HBMECs), which express high levels of ephrin-B2, whereas other endothelial cells with no detectable ephrin-B2 expression, are resistant to NiV infection [107]. Blood cells, excluding macrophages and dendritic cells (DC), in which low NiV replication has been observed, are not permissive to NiV infection, however, they can bind the virus on their surface and transport and deliver the pathogen to new sites of cell recruitment during inflammation processes [25,108,109]. This would mean that henipaviruses have ligands that allow virus binding but not replication. This was also shown when NiV was able to bind endothelial cell lectin through LSECtin ligand [98].

Ephrin receptors are receptor tyrosine kinases (RTKs) that are highly conserved amongst different species. For instance, when comparing human ephrin-B2 with murine, pig and fruit bat homologs, the receptor differs only in 3%, 4%, and 5% of amino acids, respectively. Moreover, henipavirus glycoprotein has been shown to bind to many mammalian ephrin-B2 homologs with very similar affinity [26]. Notably, ephrin-B2 is present in arterial (but not venous) endothelial cells, in neurons, glial cells, epithelial cells of the upper respiratory tract, alveolar pneumocytes, in smooth muscle cells, in macrophages in human spleen and lymph nodes and macrophages in pigs [108,110]. Differences in ephrin expression between arteries and veins are exemplified in Syrian hamsters, where NiV has been shown to be present in small and medium-sized arteries but not in veins [111]. The brain is very rich in ephrin-B2 expression, especially the prefrontal cortex and neuroepithelial cells; however, ephrin-B2 is also present at lower levels the olfactory bulb and the amygdala. In addition to the brain, the lungs, the placenta and the prostate have high levels of ephrin-B2 [112]. In contrast, ephrin-B3 is mainly expressed in the central nervous system (CNS), as well as in the heart and the prostate. The highest levels of ephrin-B3 in the brain are in the occipital lobe, the prefrontal cortex and the amygdala. However, lower levels of ephrin-B3 can be found in other regions such as the subthalamic nucleus, the pons, the temporal lobe, the globus pallidus, the hypothalamus, the hippocampus and the corpus callosum [113]. Interestingly, ephrin-B3, but not ephrin-B2, is expressed in the brainstem [114]. When infecting African green monkeys with HeV, high levels of viral antigens were detected in the brainstem [115]. Thus, although ephrin-B2 likely aids in establishing primary henipavirus infection, ephrin-B3-mediated cellular pathology increases the range of reach and pathogenicity of the viruses.

Altogether, this widespread distribution of receptors in different tissues and amongst so many hosts may explain the systemic nature of the infection and why henipaviruses have such a wide range of hosts compared to most other paramyxoviruses.

Another pathogenic mechanism of henipavirus infection is the formation of multinucleated giant cells known as syncytia. As a result of the expression of henipavirus glycoproteins on the surface of the infected cell, the viral G protein can bind to cellular receptors on neighboring cells and thereby trigger the F protein to mediate membrane fusion, thus generating a highly cytopathic effect [116]. The formation of syncytia is associated with necrosis, vasculitis and thrombosis, as well as with brain parenchyma lesions that then lead to the typical clinical signs observed during henipavirus infection, namely respiratory disorders, neurological symptoms and unstable blood pressure [27,117]. Widespread vasculitis in the lung (62%), kidney (24%), heart (31%) and the CNS (80%) has been observed during the autopsies of NiV deceased patients [27], matching with ephrin-B2 expression in these tissues [118]. Moreover, necrosis was observed in highly vascularized organs such as the spleen, where NiV was detected in the white pulp, corresponding to the only region where ephrin-B2 is expressed in the spleen [27,110]. When treating ephrin-expressing cells in vitro with HeV and NiV peptide-fusion inhibitors, live virus infection was blocked in vitro, thus suggesting a potential therapeutic possibility [119]. 

#### Henipavirus Immune Responses in Humans

The factors that render henipaviruses so pathogenic for humans are not fully understood. It is known, however, that during NiV infection, there is an increase in CXCL10 (IP-10) levels in endothelial cells and the brain of NiV-infected patients. Similar changes in IP-10 expression are seen in experimentally infected Syrian hamsters and infected human lung tissue in a human lung xenograft mouse model [120,121,122]. 

Another virulence mechanism of henipaviruses is linked to the capacity of the viral proteins C (produced from an alternate reading frame to the *P* gene), V and W (produced by RNA editing from *P* gene)*,* to antagonize the interferon (IFN) signaling response [123]. Indeed, in vivo experiments recombinant NiV strains lacking either C or V (but not W) were shown to be attenuated when infecting Syrian hamsters, resulting in 100% survival and no disease signs. Moreover, low levels of viral replication and only seroconversion was detected in animals infected with NiV strains lacking C and V, respectively [124]. In another study, while two recombinant NiV strains lacking C protein were shown to be highly attenuated, the percentage of survival rate in animals ranged between 10 and 80% depending on the virus used and the viral dose administrated [125]. This, combined with in vitro data showing that endothelial cell infection with NiV lacking C expressed more proinflammatory cytokines compared to the wild-type virus infected cells, suggests that during NiV infection, C protein helps to prevent the control of the infection by inhibiting the early proinflammatory response of the host [125]. The fact that IFNAR-KO mice lacking the type I interferon receptor are susceptible to NiV infection but not wild-type mice, confirms the role of the innate host immune response in NiV pathogenesis. 

As mentioned above, NiV *P* gene encodes 4 viral products: P, V, W, and C. P and C are generated from two different ORFs in the *P* gene; C is translated via an alternative translational start site of the *P* gene. The unedited *P*-gene transcript encodes the P protein; in contrast, V and W are synthetized when there is mRNA editing in -UC rich regions of the *P* gene by adding one single G and two G nucleotides, respectively, at the editing site [126,127]. All four of these *P* gene proteins antagonize IFN signaling; when V protein is in the cytoplasm, it binds STAT1 and STAT2, preventing the dimerization process and thus inhibiting their transport to the nucleus to activate *ISG* genes. Likewise, NiV-W protein has a similar role; however, it sequesters STAT1 in the nucleus to inhibit subsequent ISG activation. P protein can also bind and sequester STAT1 in the nucleus, but this is done to a lesser extent than V and W proteins [128,129]. C protein exerts IFN antagonist activity in the cytoplasm, but the details of this inhibition are still not well known; so far, it is only known that C interferes to some degree with RNA synthesis and that it can bind Inhibitor of κB kinase α (IKKα), thus antagonizing TLR7/9-dependent IFN-α induction [130,131,132,133]. 

In addition to STAT1 inhibition, other IFN antagonism mechanisms are seen during NiV infection: NiV-W blocks IFN signaling through both TANK-binding kinase 1 (TBK1) and Inhibitor of κB kinase ε (IKKε) [134] while V protein can inhibit STAT2 [135], LGP2, RIG-I [136], and MDA5 [137], thus preventing downstream signaling. Likewise, NiV-W protein locates in the cellular nucleus where it can interfere with activated phosphorylated forms of IRF 3, thus reducing the activation of IFNβ promoter, one of the regulators of IFN type I production [124]. These mechanisms of IFN inhibition seem to be conserved amongst henipaviruses; similar to NiV, it has been shown that the HeV-V protein can also inhibit IFN responses when it binds both STAT1 and STAT2 in the cytoplasm [138].

Interestingly, the non-pathogenic henipavirus CedV also encodes both the viral P and C proteins but lacks an alternative coding sequence for V or W. Whilst both P and C have also been shown to counteract innate immune activation, in keeping with this observation, CedV has been shown to be unable to antagonize STAT proteins, thus suggesting that the ability of NiV to cause severe disease in humans is due, at least in part, to its ability to antagonize the IFN response via viral V and W proteins [139,140]. 

Not only *P* gene products have been shown to inhibit interferon responses during NiV infection. In fact, NiV matrix protein (NiV-M), which is known to have a role in NiV assembly and budding, can also inhibit IFN-I. During infection, NiV-M protein interacts with TRIM6, thus promoting its degradation, reduced polyubiquitination of IKKε, and consequently, reduced IFN-mediated responses [141]. A further viral protein involved in inhibiting IFN-dependent responses is the nucleoprotein N of both NiV and HeV that can inhibit type I and II IFN responses. It has been shown that they hamper STAT-complex formation thus reducing STAT nuclear accumulation and consequent ISG activation. Moreover, NiV-N was shown to also directly prevent STAT import to the nucleus [142].

**Figure 3:**
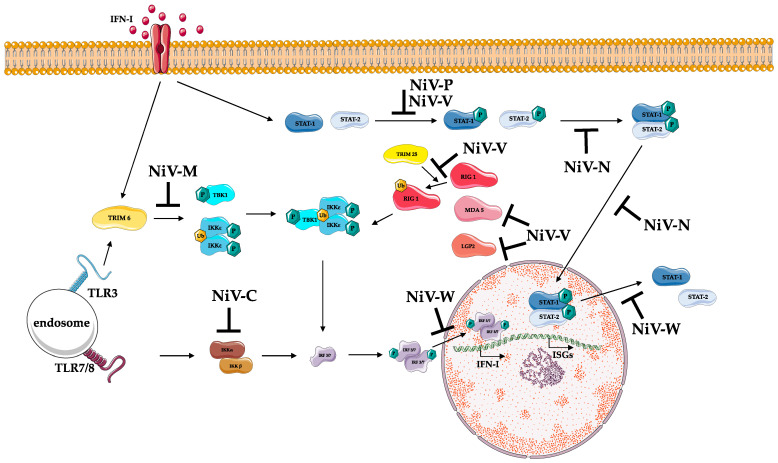
Schematic representation of NiV innate immune modulation in humans. NiV uses several immune modulation mechanisms to alter type I interferon (IFN-I) production and signaling. TLR and RLR detection of NiV RNA leads to IFN-I and IFN-stimulated gene (ISG) activation; however, several NiV proteins interfere in this process at different levels: NiV-V can prevent RIG-I, MDA5, and LGP2 stimulation. Conjointly with NiV P, NiV-V can also prevent STAT phosphorylation. NiV-N can inhibit STAT dimerization and also its nuclear importation. In addition, NiV-W prevents STAT1 and STAT2 nuclear exportation. NiV-M induces the degradation of TRIM6, thus preventing IKKε ubiquitination (Ub), oligomerization, and phosphorylation (P). NiV-C inhibits IKKα/β dimerization, necessary to activate IRF3 and IRF7, and in the same pathway, NiV-W protein inhibits nuclear transport of phosphorylated IRF3/7 dimers. Altogether, these mechanisms prevent the expression of IFN-I and ISG genes.

The main NiV inhibitory mechanisms of the immune system are displayed in Figure 3.

With regards to the adaptative immune response to henipavirus infection in humans, there is currently not much information available as to whether this response is either inefficient at dealing with the virus or if the immune response is itself detrimental for the host, thus exacerbating the pathogenic process. During NiV infection, IgM and IgG were present in high levels at day 1 and day 25 post-admission, respectively [143]. Moreover, it has also been observed that while absolute numbers of T lymphocytes in NiV infected patients remain within normal levels, a marked elevated activation of CD8 T cells (HLADR+/CD38+) was detected. In addition, proliferating CD8 lymphocytes were shown to express high levels of PD-1 and granzyme B, markers of acute effector cells [144]. In the case of HeV patients, it has been shown that reactive IgM and IgG were maintained 18 months after HeV infection and in one case, HeV reactive IgG levels were maintained up to 6 years [145].

In general, once henipavirus primary infection has taken place, the inflammatory response generated due to virus replication leads to systemic infection with more generalized symptomology. Cellular dissemination of the virus can take place in several ways: (i) replication in endothelial cells facilitates viremia and the dispersion of the virus through the blood stream; (ii) when replicating in neurons it can spread through the CNS; (iii) through disruption of the blood–brain barrier (BBB) when it replicates in endothelial cells; (iv) through syncytia formation; (v) by attaching to blood cells without infecting them, thus delivering the virus to other tissues; or (vi) through olfactory neurons from the nasal cavity [88,146,147].

### 2.3. Pathogenesis in Accidental Hosts

#### 2.3.1. Pathogenesis in Horses

As mentioned in earlier sections, no clinical signs have been detected in HeV or NiV naturally infected bats. However, this is not the case in other hosts. An example of this is horses, where HeV has a fatality rate of 90% and infection results in fever, depression, and respiratory and neurological disease [148]. Due to the difficulty of working with large animals in Biosecurity level 4 (BSL4) facilities, very few experiments have been performed in horses. Experimentally HeV-infected horses mimic the clinical signs of infection seen in horses with natural infection; the main tissue lesions observed included interstitial pneumonia and systemic vasculitis and viral RNA shedding was detected in urine and nasal and oral swabs starting before the onset of disease signs, meaning that asymptomatic horses could spread HeV [89]. Due to the number of HeV infections in horses and the presence of HeV in stalls, the most likely route of HeV transmissions in horses seems to be through very close contact [149].

#### 2.3.2. Pathogenesis in Pigs

In cases of natural spillover, infection with NiV has mostly affected pigs and humans. In the case of pigs, it has been observed that the severity of the disease depends mostly on the age of the animals. While mortality can reach 40% in suckling piglets, pigs that are over 4 weeks old rarely succumb to the disease, with only 1–5% of animals dying from infection [150]. In pigs, NiV causes a disease known as porcine respiratory and encephalitis syndrome (PRES) and mostly consists of an acute fever with respiratory signs characterized by difficulties in breathing, a barking cough, also known as barking pig syndrome (BPS), nasal discharge and in some cases neurological signs [14]. Despite affecting the respiratory system in both humans and pigs, the respiratory symptoms observed in pigs are much more severe. Experimentally infected pigs show either subclinical signs or respiratory and neurological signs with interstitial pneumonia, systemic vasculitis and meningitis, similar to what has been observed in naturally infected pigs. In terms of transmission, NiV has been detected through viral isolation in the nose and the throat of both symptomatic and asymptomatic pigs and importantly, infected pigs are able to transmit NiV to naïve pigs several days post NiV inoculation [17,146]. All this suggests that probably, pig-to-pig transmission of NiV requires close direct contact with nasal secretions from symptomatic or asymptomatic NiV-infected pigs.

#### 2.3.3. Pathogenesis in Humans

Both, HeV and NiV are very pathogenic for humans and have a fatality rate that can reach 60% and 92%, respectively [1,2]. Disease symptoms normally appear from day 3 to 14 for NiV and 4 to 16 for HeV post exposure and in humans are characterized by high fever, respiratory symptoms and in some cases neurological disease with long-term symptoms; in 3–7% of infected patients relapse is possible months to years after the initial infection [151,152,153]. Infected humans frequently showed interstitial pneumonia, systemic vasculitis and in some severe cases, meningitis [27]. In terms of transmission, HeV has never been proven to be transmitted to other humans; however, NiV is easily shed in urine and respiratory secretions and is significantly transmittable amongst humans (see previous section of transmission). 

In natural outbreaks of NiV and HeV disease, the oronasal route seems to be the main route of infection. During NiV infection, epithelial cells and type II pneumocytes from the bronchiole are the primary targets [39] and infection induces the production of inflammatory cytokines such as IL-6, IL-8, IL-1α and granulocyte-colony stimulating factor (G-CSF), responsible for the recruitment of immune cells. This phenomenon may lead to the development of an ARDS-like disease [154,155]. 

**Figure 4:**
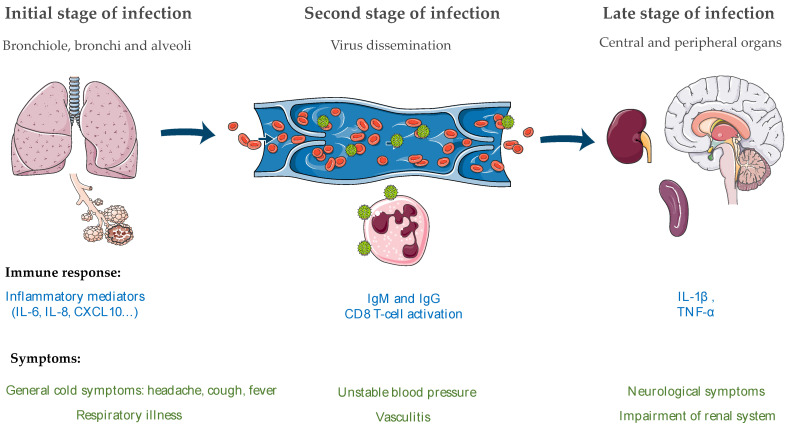
Henipavirus pathogenesis in humans. (**left**) During the initial state of henipaviruses infection, the respiratory tract and specifically the bronchi and alveoli are affected. Inflammatory mediators are released in consequence; (**middle**) In a second stage, the virus is systemically disseminated through the blood stream, either freely or attached to the surface of leukocytes; (**right**) In the late stage of infection, the virus reaches several organs such as the spleen, kidney and central nervous system (CNS), where the blood–brain barrier (BBB) is disrupted and cytokines such as tumor necrosis factor (TNF)-α and IL-1β are released, ultimately leading to the development of neurological signs.

As shown in Figure 4, after infection of the epithelium in the lung, the virus spreads to endothelial cells and eventually will gain entry to the blood stream where it will disseminate to other tissues, thus leading to a possible organ failure of the lungs, spleen, kidneys and brain [155]. The virus can also enter the CNS either through blood vessels of the brain and/or through olfactory nerves [146], which will disrupt the BBB and then induce IL-1β and tumor necrosis factor (TNF)-α production that may lead to development of neurological signs. NiV inclusion bodies and necrosis can in some cases be found in both the gray and the white matter [155]. The disruption of the BBB is believed to be due either to the expression of TNF-α and IL1-β by microglia or other surrounding cells or to the direct cytopathic effect of NiV replication in the microvasculature.

Concerning HeV, while the exact route of transmission is not known, it has been observed that the respiratory tract is one of the first replication sites of the virus [156]. The infection of both the upper and lower respiratory tract induces the release of inflammatory cytokines including IL-6, IL-8, IL-1α and MCP-1 that contribute to the pathological changes observed during HeV infection, such as respiratory distress, the appearance of severe pulmonary edema and vascular injury. Additionally, while it is not well known how HeV spreads to the CNS, there are some neurological signs observed during infection such as encephalitis and drowsiness [155]. 

Likewise, during HeV infection in vitro, in small airway epithelial human cells, inflammatory cytokines have been shown to be released. Of interest, it has also been shown in vitro that HeV is less efficient in counteracting IFN compared to NiV Malaysia and it may be speculated that this could partially contribute to the lower mortality rate of HeV in humans when compared to NiV [122]. 

## 3. Immune Responses in Bats as Reservoir Hosts for Henipaviruses

The immune system consists of several components including (i) physical and chemical barriers and (ii) immune effector humoral and cellular mechanisms [157]. These components, despite being highly conserved amongst vertebrates, differ enough between species to generate variable susceptibility to pathogens. As discussed above, bats are important reservoir species for many virus families with known potential to cause disease in humans and livestock species. In this section, we will present what is known about henipavirus infection in bat species, in both cases of natural and experimental infection, and also present an overview of what is currently known about the bat immune responses to viral infection and the similarities and differences in these responses compared to humans. 

### 3.1. Biological Factors

Although much information has been collected about bat ecology and physiology, relatively little is known about their immune system. Interestingly, despite the fact that bats are persistently infected with many viruses, they rarely display clinical signs [41]. This is also the case for HeV and NiV, that have clearly co-evolved with their natural hosts and at least for HeV appears to require a susceptible intermediate bridge species in order to emerge in humans [158]. However, little is known about the effects of henipaviruses in Pteropus bats and the factors responsible for maintaining henipavirus infection among bat populations and the immunological mechanisms involved are still not well characterized. Several experiments have shown that experimental infection of bats with HeV and NiV leads to seroconversion but no clinical signs are observed [68,159]. In terms of viral spread, it has been observed that mucosal viral replication is followed by systemic viraemia during HeV infection in bats [68]. However, the viremic phase of HeV and NiV infection in infected bats is quite short, since it is possible to isolate both viruses from blood at early points (7 days following infection) after inoculation, but not always at later timepoints post-infection (p.i.) [159,160]. During experimental infection of bats with henipaviruses, it has been observed that shedding occurs rarely, in only a few animals, at very low titers, and in narrow time frames after infection [68,159]. This also seems to be the case in naturally infected bats [39,161]. For HeV, experimentally infected pregnant and lactating females have been shown to have a higher seroprevalence compared to males for some bat species but not others [68,162]. The high seroprevalence of HeV in bats in nature suggests bat-to-bat transmission, but experimental attempts have yet to prove it [160]. Even if more studies are required to determine the factors that aid HeV and NiV bat-to bat transmission, it seems clear that it requires direct contact and that the high density of their colonies and the use of urine in grooming may contribute to the spread of the virus amongst roosting individuals [159].

Interestingly, with the exception of lyssaviruses (such as rabies virus), bats and bat-borne viruses coexist and indeed bats appear disease-free from most of the emerging or re-emerging pathogenic viruses that cause disease in humans [163]. Some factors that could contribute to minimize viral pathogenicity in bats may be related to their high metabolism rate, specific inflammation conditions, differences in body temperature and constitutive immune factors amongst others [164,165]. The route of infection may also have an important repercussion in the outcome in bats; naturally infected bats are believed to remain healthy and seroconvert [166]. For instance, for rhabdoviruses, it has been shown that experimentally infected bats can be rendered susceptible to the disease depending on the route of infection: while intracerebral infection leads to death, intramuscular infection caused disease in only 30% of the experimentally infected individuals [167,168,169]. 

Despite the diversity and sheer number of viruses harbored by bats, they are not considered to cause high numbers of fatalities or to reduce their lifespan. Bats live much longer than similar-sized mammals and outlive even birds [170]. This great longevity together with their dense roosting ecology represent a potential opportunity for long-term viral persistence in a bat population over several generations [171]. Another feature in bats that may contribute to virus persistence is the fact that they can fly. This is special not only for the fact that the plasticity of their wings allows them to extend the ecosystems that they can inhabit but this action of flying requires that they have a very high concentration of red blood cells and that they consume four times more oxygen during flight compared to when they are at rest [172]. Moreover, flying requires extremely demanding metabolic mechanisms which may explain why bats contain an enrichment of mitochondrial and nuclear-encoded oxidative phosphorylation (OXPHOS) genes involved in metabolism in comparison with background genes [173]. It has previously been shown that oxidative stress may have an impact not only on the host but also on the pathogens that they contain [174]. In fact, the upregulation of oxidative stress has been shown to decrease the survival and spread of pathogens [175]. All of these evolutionary adaptations may have had repercussions on bat metabolism and affected bat immunity and their capacity to harbor viruses in a non-pathogenic way. This may translate to differences in immunity that contribute to their ability to combat virus infection. 

### 3.2. Immune Factors

When looking at the high number of viruses that coexist with bats without rendering them sick, it seems like bats have developed more efficient mechanisms to control viral replication compared to other mammals. This is also the case in rodents that frequently do not show clinical signs in response to the viruses that they carry [176,177]. 

All viruses have immune-modulating genes that provide at least partial protection from the host immune response. If successful, they may evade the hosts’ immune defenses thus allowing replication and transmission to other potential hosts. One of the hypotheses to explain bat resistance to pathogens is that they may control viral replication very early during infection. Studying viruses from a non-pathogenic perspective has not frequently been done due to the lack of models and tools, but it is crucial to extrapolate the mechanisms that protect bats from clinical infection. Here we review the different components of the bat immune system.

#### 3.2.1. Immune Cell Populations

Despite a lack of reagents to study bat specific cell types, certain immune bat cell populations have been described through morphology and physiology studies using techniques such as electron microscopy and cell adherence assays amongst others. Notably, T and B lymphocytes, cells resembling follicular dendritic cells, macrophages, neutrophiles, eosinophils and basophils have been so far identified and look similar in terms of morphology and ratio compared to those observed in mice and humans [178,179]. Importantly, a bat–mouse marrow chimera has recently been generated that reproduces a bat’s biological systems. In this model, to solve the limitation in bat-reactive antibodies that exist, the authors used mice and human antibodies that showed cross-reactivity with other species [180]. 

#### 3.2.2. The Innate Immune Response

##### Pattern Recognition Receptors

Pattern recognition receptors (PRRs) are proteins capable of recognizing molecules typically found in pathogens. There are mainly two types: Toll-like receptors (TLRs) and cytosolic retinoic acid inducible gene-like helicases (RLHs). There are 13 TLRs described in mammals (11 in humans) and they are highly conserved amongst species [181]. TLRs are mainly expressed in DC and macrophages. For example, TLR3 detects double-stranded RNA, TLR7 and TLR8 recognize single-stranded RNA, and TLR9 binds unmethylated cytosine-phosphate-guanine motifs. Importantly, only TLRs 3, 7 and 9 are involved in interferon type I induction during viral infection [182]. TLRs from 1 to 10 and 13 have been shown to be present in *P. alecto* bats but TLR 13 showed stop codons within its open reading frame meaning that it may be a pseudogene. When a transcript is obtained for a pseudogene, normally it indicates that this gene has only recently undergone inactivation and that before that, it would code for a protein with a specific function [183]. Other than in bats, the only mammal where TLR13 has been found is in rodents, which is very interesting since both species are resistant to many human pathogens [184]. Among the known TLR types, TLRs 3, 7, 8 and 9 participate in virus detection. It has been shown in *E. fuscus* bat cells, for example, that TLR3 is able to detect exogenous double stranded RNA; however, binding assays to determine the ligands of this receptor have still not taken place [185]. Despite the fact that this study was not performed in bat cells, it has been shown that during NiV infection of human 293T and Hela cells, NiV-W secreted protein can directly inhibit the TLR3 pathway [134]; differences between humans and bats in terms of the TLR3 ligand domain or in terms of events occurring downstream of the TLR3 pathway could maybe explain discrepancies in innate immune interactions and detection of henipaviruses between species.

There are three members of the RLH detectors found in mammals: retinoic acid-inducible gene I (*RIG-I*), melanoma differentiation-associated gene 5 (*MDA5*), and laboratory of genetics and physiology 2 (*LGP2*). RIG-1 and MDA5 have been detected in most bat genomes and, together with LGP2, all have been shown to be present in *P. alecto*. RIG-I and MDA5 in *P. alecto* share their primary structure and functionality when compared to their human and rodent homologs and patterns of tissue expression are comparable to their human counterparts. *P. alecto* kidney cells produce IFNs when stimulated with poly(I:C) and RIG-I and MDA5 can sense poly(I:C) in *E. fuscus*. Thus, both membrane and cytosolic RNA sensors are conserved and functional in bat cells [183,185,186,187]. It is known that NiV dsRNA is sensed by RIG-I, but not by MDA5, and that this detection can initiate an IFN response [188]. However, it has been shown in vitro that NiV-V can bind to MDA5, thus antagonizing antiviral immunity [189].

For HeV, it has very recently been shown that in experimentally infected black flying foxes, viral antigen is detectable in the lungs 60 h post-infection and that there is a considerable increase in type I and II IFN and CXCL10 release in both the lungs and the spleen of these HeV-infected bats. Moreover, liquid chromatography tandem mass spectrometry (LC-MS/MS) analysis of HeV infected lungs from these animals showed upregulation of cell mediated immunity and enrichment of the type I IFN signaling pathway [190].

Therefore, despite having proven that bats have as many functional TLRs and RLH as other species of mammals, there have still not been enough studies about their potential interaction with bat-borne viruses including NiV and HeV. It can be hypothesized that some differences can still remain at the signaling pathway level which may lead to differences in the early detection of NiV and HeV infection in bats. 

##### Interferon and Soluble Immune Mediators

The IFN response is one of the first and most powerful lines of defense against viral infection. There are three types of IFN described so far: types I, including α, β, ε, ω, κ, δ, τ, and ζ), II (IFN-γ), and III (which includes IFN λ), which differ in their sequences, their receptors and the cells that produce them. Type I and III IFNs play an important role during innate immunity since they are directly induced during the early stages of viral infection. Even though their receptor is different, in the end, they both induce IFN-stimulated genes (*ISGs*) which led directly to the antiviral activity of IFNs [191]. In humans, there are 13 IFNα genes but in bats only 7 IFNα genes have been identified in *P. vampyrus*, 7 genes and 1 pseudogene in *D. viridis*, and only pseudogenes in *M. lucifurgus*. Both *M. lucifugus* and *P. vampyrus* also have around 12 IFNω in contrast to humans that only have 1 functional IFNω and at least 2 pseudogenes [192]. Thus, the expansion of the IFNω family in bats could have implications for antiviral immunity. Type III IFNs have also been detected in *M. lucifugus* and *P. alecto*. Importantly, type III IFN has been shown to have a wide distribution, contrary to humans, thus maybe indicating a more significant antiviral role of IFN in bats [193,194,195].

IFN production pathways have been shown to be functional during both in vitro infection in bat cells and in vivo [196,197]. Viruses can antagonize either IFN signaling or IFN production pathways, sometimes even both. However, this capacity for inhibition is different between not only viruses but also between hosts (bats versus humans for instance) [194,198]. In *P. alecto* splenocytes, for example, type I IFN is downregulated and type III IFNs upregulated during infection with the paramyxovirus Tioman virus. In contrast, henipavirus infection downregulates both type I and III IFN production in human cell lines and IFN production and signaling in Pteropid bat cell lines [198,199,200]. Evasion of both the signaling and the production pathways of IFN in bat cells may indicate that other antiviral factors play a role in bat immunity. However, it should be taken into account that the wide diversity in bat species may represent important molecular differences in immune mechanisms, thus making it difficult to achieve a homogeneous view of immune responses in bats.

In general terms, the interferon response in cells starts when viral genomes are detected by pathogen pattern recognition receptors. Thus, during henipaviruses infection, viral RNA is detected by endoplasmic sensors such as TLRs 3, 4, 7, and 8 and by cytoplasmic sensors such RIG-1 and MDA-5. When viral RNA binds with TLR3, TLR adaptor molecule 1 (TRIF) will mediate downstream signaling that then induces IFN production. In the case of viral RNA binding to either RIG-1 or MDA-5, a signaling cascade is triggered that causes phosphorylated IRF3 and/or NFκB translocation into the nucleus where they lead to type I (IFNα, IFNβ) and type III (IFNλ) IFN synthesis.

Production and signaling are both very important to start an IFN response during viral infection. However, the signaling pathway in bats has not been well explored. In general, when IFN binds to its cell surface receptors, it activates the JAK-STAT pathway which will then lead to the phosphorylation and activation of the STAT family of transcription factors. Phosphorylated STAT1 and STAT2 form dimers and bind to IFN regulatory factor 9, thus translocating to the nucleus and finally inducing ISG production and activation of an antiviral state [201]. Currently, only the STAT1 protein has been characterized in bats [202,203]. In these studies, it has been shown that the bat STAT1 signaling pathway is similar to that found in other mammals. However, characterization of other signaling molecules of the IFN response may help to further understand innate antiviral immunity in bats.

The IFN response in *P. alecto* has different kinetics compared to that observed in human cells. In all species, the IFN response is under strict regulation. The reason for such a strict regulation may be to avoid an exacerbated inflammatory response; it has been observed that constant activation of IFN can lead to high levels of inflammation that can be detrimental for the host. Bat ISGs showed similar early induction kinetics to humans, however they decline in a late phase. In contrast, in human cells, ISGs remain elevated for longer periods. Moreover, in bats, IFN was able to induce the antiviral effector 2-5A–dependent endoribonuclease, which contributes to viral control but that in humans is not an ISG [204]. Such differences in kinetics may be crucial to control virus replication without causing damage to the host during an immune response. 

Cytokines play an important role during viral infection since they encompass and regulate the components of the immune system. However, a dysregulated excessive inflammatory response can be detrimental, cause tissue damage, and lead to morbidity and mortality of the host [205]. 

Many bat cytokine genes have been characterized including interleukin (IL)2, IL4, IL6, IL10, IL12p40, IL-23a, tumor necrosis factor (TNF) and the granulocyte macrophage colony-stimulating factor [206,207]. These cytokines are highly conserved when compared to other mammals [194]. It is believed that bats have also evolved mechanisms to avoid excessive inflammation. Some studies have shown that stimulation of bat cells with polyinosinic-polycytidylic acid (polyI:C) leads to a reduced pro-inflammatory response compared to human cells whereas a robust TNFα response was displayed. A TNF response is controlled by several transcription factors, amongst them the NF-κB pathway, which is a major transcriptional regulator of inflammation. There are five members of the NF-κB family: RelA (p65), RelB, c-Rel, NFκB-1 (p50) and NFκB-2 (p52). Of these, c-Rel has undergone positive selection in the bat ancestor [208]. There is further evidence of bat specific adaptations in genes involved in antiviral and pro-inflammatory signaling. When compared to other mammals, RIG-I, IL1b, IL-18, NLRP3, STING and CASP1 pathways contain adaptations associated with reduced inflammatory responses in bats [209]. Thus, bat cells seem to mount a strong antiviral cytokine IFN response but a low inflammatory response that allows the control of many viral infections. The balance between resistance and tolerance may be attained through very specific selection of the pathways that are activated and shorter periods of activation to prevent inflammation.

##### NK Cells

Despite the fact that NK cells are important players in the antiviral immune response, a transcriptomic study in bat tissues showed less NK cell-related gene coverage compared to other mammals. Importantly, the *R. aegyptiacus* genome has been shown to have a different repertoire of NK cell receptors and lacks functional killer cell immunoglobulin receptors (KIRS). Moreover, in this study, all killer lectin-like receptors (KLRs) were shown to have either activating and inhibitory interaction motifs, or only inhibitory motifs [210]. This is different from other mammals where NK cells normally possess both stimulatory and inhibitory receptors. Understanding the importance of these differences in terms of viral recognition and containment requires further study.

#### 3.2.3. Adaptive Immune Response

Several studies have proven that bats have both cellular and humoral immune responses; however, the maintenance and generation of these responses was shown to be different not only from other mammals but also amongst bat species. In this section, we will explore these differences.

##### Antibodies

Antibodies are Y-shaped molecules secreted by B cells. The two arms are involved in binding the antigen and contain the variable (V) region; the stem of the antibody (C region) is less variable and is involved in interacting with effector cells of the immune system [211]. There are five classes of antibodies: IgA, IgD, IgE, IgM and IgG that differ in their C region. All antibody classes have been described in bats: at least four species of bats (*Carollia perspicillata*, *M. lucifugus*, *E. fuscus* and *Cynopterus sphinx*) have been shown to transcribe IgM, IgA, IgE and several IgG classes [212]. However, IgD, which is not present in all mammals, seems to be present in microbats such as *M. lucifugus* but not in megabats. A wider screening in megabats should be done in order to confirm that this is the case [213,214].

In terms of antigen recognition, both microbats and megabats have been shown to have a highly diverse antibody repertoire, as rich as humans and mice and richer than in most other mammals [215]. The variable region of *P. alecto* has been shown to be rich in tyrosine amino acids when compared to other antibody mammals [216]. Tyrosines confer structural diversity and are implicated in antigen binding [217,218], which could explain differences in antibody binding capacity and the simultaneous presence of several viruses and antibodies in the same individual; however, further characterization of these interactions is required [219].

There is an insufficiency of henipavirus-associated immune dynamics data in natural infection; this includes the duration of immunity, differences between gender, adults/juveniles, in free-ranging pteropid bats, etc. NiV and HeV experimental infection in Pteropus bats have been shown to elicit an antibody response following infection [68,149,159]. In nature, the serostatus in females was shown to be related to seasonality; the peak of rain season and the end of gestation is the moment where NiV antibodies are highest in Madagascan fruit bats [69]. Moreover, several studies have suggested a reduction of henipavirus antibodies in pteropid juveniles probably due to the loss of maternal antibodies over their first year [162,220,221]. This indicates that bats may seasonally control pathogens. However, in other studies in Bangladesh and surrounding areas, it was shown that viral dynamics are not annual or seasonal but they are cyclical and they are driven by demographic and immunological factors [221]. These differences between studies could be due to differences between bat species or lack of comparable ecologic factors. 

When compared with other mammals, bats present quantitative and qualitative differences in antibody responses. Moreover, there is also variability between species and viral infections [164]. Antibody responses can differ depending on the kinetics and the magnitude of the response. Certain bat species show a delay in attaining the peak of the primary antibody response when compared to other mammals [222,223]. Secondary antibody responses with IgG have also been shown to be slower or inexistent. However, a higher affinity of the antibodies in bats has also been proven [223,224]. This could be explained by either the existence of a higher innate affinity in bats or a higher IgG maturation due to the repeated exposure to certain pathogens.

Providing long-lasting protection is one of the hallmarks of the adaptive immune response. Even if vaccination of bats seems to protect them from infection, the fact that antibodies against the pathogen are not always detected suggests that the protective immunity mechanisms in bats may differ from other mammals. Thus, the failure to detect specific antibodies is not enough to exclude prior exposure. Moreover, it has also been shown that changes in virus titers during NiV infection in *P. vampyrus* were also linked to changes in the neutralizing antibody response, thus meaning that maintenance of virus in bats does not always sustain an antibody response [69,225]. In nature, NiV transmission seems heightened when there is a waning in the seroprevalences of NiV IgG antibodies in bat individuals or a decrease in herd immunity [220,221], thus suggesting a limited duration of both individual and herd immunity. This is supported by a more recent study where henipavirus infection in fruit bats was shown to be recurrent and their immunity, if present, was estimated to last between 1 and 2 years [226].

Another difference in bats immune systems lies in the fact that they have more expressed surface immunoglobulin (∼82%) compared to humans and mice (∼15–30%) [227]. In order to determine the nature of these populations, further not-yet available bat-specific reagents are needed.

##### T-Cell Responses

T lymphocyte-cell responses include cytotoxic and helper functions. To date, only T-cell coreceptor CD4 has been characterized in bats [228]. However, T-cell responses have been described, and are indicated to be slower, having a peak at 120 h post infection compared to 48 h in mice [229,230]. This delay was also observed in mixed lymphocyte responses (MLR) where the peak for *Pteropus giganteus* bats was observed at day 7 compared to day 5 in mice. This indicates that in bats, cell-mediated immunity is slower compared to other mammals [222]. MLR tests are normally used to test the recognition and proliferation of T cells from different individuals, basically, T cells from one donor will proliferate in the presence of antigen-presenting cells from a different donor and this response is highly dependent on MHC class II polymorphism [231]. Another study in *Noctilio albiventris* bat lymphocytes has shown that when studying *MHC DRB* locus diversity (the exon that encodes the peptide-binding region of MHC), significant differences were seen in MHC genes polymorphism [232]; moreover, this polymorphism seems to have been influenced by pathogen-driven selection [233]. In a different study, females showed lower MHC heterozygosity than males, thus meaning that the selection pressures acting on the *MHC* gene may differ between sexes [232]. Such differences may influence the ability of different populations of bats to respond to infection.

### 3.3. Conclusions

Functional and genome sequence analyses of bats have revealed that bats share many of the immunological features of other mammals and that they have similar cell populations and activation pathways. However, differences in kinetics and in levels of expression may be crucial for the control of viral replication.

Although bat cell line studies may help to shed light on several mechanisms, important differences are found between in vitro and in vivo bat data. For instance, while *R. aegyptiacus* cell lines are equally susceptible to Marburg virus (MARV) and Ebola virus (EBOV) [234], infections of *R. aegyptiacus* seem to confirm that this species is a reservoir for MARV but that this is probably not the case for EBOV, since infection resulted in very low viremia, low replication in other tissues, and no viral shedding. In contrast, MARV virus infection resulted in high viremia and dissemination to other tissues [235]. Another such difference seen in in vitro studies is the fact that despite not detecting type I IFN in *R. aegyptiacus* [234], type I IFN is induced in *R. aegyptiacus* cell lines during Sendai virus infection [210]. 

One of the limitations of bat immunology studies consists in the difficulty in working with bats in terms of capture and husbandry. Furthermore, while bats seem similar, they are physically, physiologically, and genetically very different between species and the fact that the majority of studies have been done with only two species (namely *P. alecto* and *R. aegyptiacus*) does not provide a clear general idea of bat immunity mechanisms. The lack of bat-specific immunological tools currently considerably limits mechanistic studies of bat immunity. However next-generation sequencing studies, the potential generation of bat organoids, and the use of the new in vivo bat chimeric mouse model may allow the undertaking of novel and important functional studies. 

## 4. Animal Models

Several animal models exist for the study of NiV and HeV pathogenicity and innate immune responses. Here we summarize the most commonly used since the discovery of these pathogens. A summary of the different animal models for HeV (in blue) and NiV (in green) can be found in Table 1.

### 4.1. Henipavirus Infection in Cats

During the NiV outbreak in Malaysia, as well as in pigs and humans, signs of infections were observed in several other animal species, including dogs, cats and horses. Cats were first used experimentally as a model for HeV infection to study transmission and the pathology of associated disease [52]. In cats, HeV infection results in similar symptoms to that seen in horses with the most severe clinical manifestations occurring in the lungs. Similar symptoms are seen with NiV infection in this animal and severe cases are associated with extensive inflammation and the presence of viral antigens in respiratory epithelia. Cats infected either by subcutaneous, intranasal or oral routes display clinical symptoms within four to eight days [236,237]. All routes of experimental inoculation result in infection and clinical illness or death, with detectable virus found in the lungs, spleen, kidneys and brain, but also in tissues such as the trachea, liver, lymph nodes, rectum, urine, bladder, heart and blood. Necroscopy confirms the observation that HeV infection in cats is primarily a respiratory disease; pathology is found mostly in the lungs, hydrothorax and pulmonary edema are associated with congestion and intrapulmonary hemorrhaging, bronchial and mesenteric lymph nodes were enlarged, pale and presented petechiae. The spleen was also found to be enlarged in infected animals. Other pathological signs included highs numbers of alveolar macrophages, alveolar wall necrosis and vascular lesions including thrombosis, necrosis and endothelial syncytia [237]. Cat-to-cat transmission from infected animals has been demonstrated [236]. 

Both natural and experimental infection of cats with NiV is possible [17,240,306]. Infection studies with either intranasal [17] or subcutaneous [238] inoculation resulted in clinical symptoms similar to those observed with HeV infection, including fever, depression and rapid or labored breathing. Autopsy and histology showed similar results to those seen with HeV. Vertical transmission of NiV has also been observed in a pregnant cat with virus isolation possible from the placenta, fetal tissue and uterine fluid after necropsy [239]. 

#### Henipavirus Immune Responses in Cats

In vaccine models, cats were shown to develop good IgG and IgA responses to NiV [306,307]. Other than those gleaned from vaccine studies, there are few data on specific immune responses to henipavirus infection in this species. 

### 4.2. Henipavirus Infection in Dogs

Several serological studies have confirmed field observations that dogs were frequently infected with NiV during the first outbreak of this virus in Malaysia [46,243,244]. Cases of infection were most probably caused by direct contact of dogs with infected pigs or ingestion of pork products. Autopsy data of two affected dogs showed pulmonary edema, interstitial pneumonia and signs of meningitis. One of the dogs displayed clinical signs similar to canine distemper. Data on the immune response to henipavirus infection in canines are lacking.

### 4.3. Experimental Henipavirus Infection in Ferrets

The immune background of ferrets is not fully understood and there are limited ferret-targeted reagents with which to perform immunological studies. However, in the same way as for hamsters, ferrets have been widely used to study henipavirus infection and transmission due to similarities in disease symptoms and progression. Ferrets infected with both NiV and HeV develop respiratory and neurological symptoms. Clinical symptoms include fever, cough (with NiV) and nasal discharge, as well as neurological symptoms including depression, paralysis or tremors [245,248,251].

Pathological findings include histopathological lesions with systemic and bronchial vasculitis and necrotic lymphadenitis. Immunostaining reveals the presence of viral antigens in multiple organs, neurons and endothelial cells of the bronchoalveolar epithelium [250]. Meningitis can be observed in animals with neurological symptoms [248]. Virus was found by quantitative PCR (RT-qPCR) in all body fluids tested including blood, from rectal swabs and urine [245,248,251]. A recent study aimed at assessing immune gene expression profiles in young ferrets infected with different henipaviruses strains found that profiles in lungs and brain tissues displayed an upregulation in macrophage markers such as CD40 and CD80. In this study, following infection of the upper and lower respiratory tract, the virus was found to spread quickly throughout the animal to other organs, with virus found in the trachea, CNS, lung, liver, spleen, heart, kidney, bladder and blood 5–7 days following infection.

#### Henipavirus Immune Responses in Ferrets

Characterization of the host response in the lung showed early activation of interferon responses and an increase in mediators of inflammation but overall, an absence of lymphocyte activation. Subsequent expression of inflammation-related genes in this organ was associated with clinical deterioration [246,252].

As for hamsters, ferrets have been widely used for the assessment of antivirals and vaccination against henipavirus infection [190,245,247,248,249,250,251,253]. Similar to hamsters, such studies have confirmed the success of varying vaccination solutions against henipavirus infection in animals in providing complete and long-lasting protection against viral challenge. Indeed, one study demonstrated continuing protection against severe disease in ferrets challenged 12 months post-vaccination with only limited detectable localized replication of virus [253]. Likewise, passive immunization has been shown effective in protecting animals from disease when administered hours to days p.i. [247,248].

Of note, ferrets were also used to assess infection with CedV [308]. As for guinea pigs, animals exposed to CedV developed neutralizing antibodies against the virus (2/2 animals) as early as 10 days p.i. in the absence of clinical infection. In a euthanized ferret, evidence of virus replication was detected in bronchial lymph nodes 6 days p.i., as well as reactive hyperplasia of tonsillar lymphoid tissue and retropharyngeal and bronchial lymph nodes. Viral RNA could be detected in lymphoid tissue 6–20 days p.i., although virus isolation was unsuccessful for all PCR positive tissues [308].

### 4.4. Henipavirus Infection in Horses

In horses, HeV causes an illness associated with respiratory and neurologic signs that frequently leads to a fatal outcome. Since its emergence in 1994 in Australia, there have been 32 HeV equine outbreaks, of which 5 have involved humans after very close contact with infected horses [309]. 

The first equine experimental infection studies suggested that very close contact is necessary to transmit HeV amongst horses and even in such cases, that it is rather a rare event. In experimentally oronasally HeV-inoculated horses, transmission to other horses or to cats could not be proven; however, transmission from HeV infected cats to horses was possible. In these experiments, it was also shown that during necropsy of HeV infected horses, the kidneys and lungs appear to be particularly affected by HeV infection and both organs present very high viral titers. Interestingly, while the urine and the mouth of HeV-infected horses presented HeV titers, the nasal cavities and the trachea did not show immunohistological detection of HeV; however, this could be explained by the fact that some of the HeV administration was done subcutaneously rather than intranasally [149]. Intravenous HeV inoculation in horses showed increased vascular damage, probably related to the route of infection [254].

In a more recent study, it was shown that oronasal administration of HeV led to its continuous detection in 2 out of 3 inoculated horses in the nasal cavities from day 2 post exposure [89]. Thus, it is possible that local replication in the nasopharynx or the nasal cavities takes place before HeV spreads systemically, depending on the route of inoculation of the virus. In this experiment it was also shown that the first HeV-related signs of infection, such as fever and high cardiac rates, appeared in horses at day 5 post-challenge. The incubation period in HeV infected horses can vary from 5 to 16 days post exposure [49,89]. These first signs are rapidly followed by depression, dyspnea, visible edema in lips, face, head and neck, intermittent recumbency and loss of appetite. In some cases, HeV-infected horses exhibit neurological signs consisting of muscle twitching, depression, disorientation, hypersensibility when approached, facial nerve paralysis and restlessness amongst others [149]. In fatally infected horses, the illness lasts approximately 48 h from the first signs [48]. However, HeV is not always lethal in horses and approximately 25% of horses survive; survival is associated with the presence of HeV-neutralizing antibodies and transmission risk has shown to be higher during the terminal stages of the disease being maximally infectious at necropsy for a period of several days depending on the environmental conditions [49,149]. 

When compared to humans, in horses, the lesions seen during HeV infection are quite similar since they both share multi-systemic features in the lungs, kidneys, spleen, bladder, meninges, etc. However, it would appear that in horses, there is higher severity of damage in the lungs where edema, thrombosis, hemorrhaging, tissue necrosis and endothelial syncytia are commonly observed [89,254,255] 

In the case of NiV, it is known that natural infection can take place in horses since during the 1998 NiV outbreak in Malaysia, seroconversion in several horses and meningitis in one horse were detected [63,240]. Moreover, during the Philippines outbreak, several horses exhibited neurological signs with some lethal cases, but it was not possible to detect the presence of NiV by histopathology since samples were unavailable [257]. To date, there have not been experimental studies in horses with NiV. 

#### Henipavirus Immune Responses in Horses

Equine immunological studies are frequently haunted by small sample numbers, the difficulty to host and manipulate horses in experimental facilities, ethical concerns, exorbitant experimental costs and difficulties in having a controlled research environment. However, since some immune responses are species-specific, immune studies carried out in different mammals do not always translate to what really takes place in the horse. Few studies exist concerning horse immunology during NiV and HeV infection. However, a recent study showed that horses immunized with commercial Hendra virus vaccine (Equivac® HeV) mounted an effective immune response consistent with the presence of protective immunity against HeV in the form of virus-neutralizing antibodies [256].

A recent xenograft model where immunocompromised mice were engrafted with either equine bone marrow or peripheral blood lymphocytes (PBLs) showed that the immune cell populations were successfully engrafted in the host mice. This new model could allow the study of innate immune responses during equine infections, including for HeV [310]. 

### 4.5. Experimental Henipavirus Infection in Monkey Models

Both Squirrel Monkeys and African Green Monkeys (AGMs) are highly susceptible to fatal henipavirus infection and both the symptoms and disease course seen with infection are similar to those observed in humans [24,258,262,269]. NiV infection induces fatal acute respiratory distress syndrome and systemic vasculitis with clinical manifestations including neurological disease [263,264,266] and dyspnea [24,265,269]. As in human and animal infection with the virus, antigens can be found in a range of tissues including the brain, liver, spleen, lungs and kidney, suggesting systemic spread of pathogen. In AGMs, it has been suggested that NiV Bangladesh is more pathogenic than NiV Malaysia [59,267]. Owing to this high susceptibility to infection and the similarities in pathologies compared to humans, the AGM model has been used to assess antiviral drug treatment [258], monoclonal antibody therapy [115] and vaccine efficiency [260] for HeV and NiV infection.

#### Henipavirus Immune Responses in Monkey Models

A recent study has also assessed henipavirus infection in cynomolgus monkeys [259] compared to the AGM model. In contrast to AGMs, in macaques, inoculation with either virus caused only mild or asymptomatic infection, despite evidence of similar replication kinetics for the two primate species. In this study, several important differences were observed in terms of immune responses between surviving animals and both macaques and AGMs succumbing to infection, including abnormal innate immune signaling, cytokine release and complement activation. Infection in macaques commonly led to activation of adaptive immunity and recruitment of immune cells including of cytotoxic CD8+ T cells, Th1 cells and plasma cells. Overall, the authors suggest that in macaques and AGMs animals that survive, henipavirus infection promotes Th1 differentiation, possibly by maintaining low levels of pro-Th2/anti-Th1 IL-4 and higher levels of pro-Th1/anti-Th2 IFN-γ early in infection. Similar results were previously seen in AGMs surviving NiV infection [265,268]. Such observations may represent an important step in understanding host immune responses to these lethal viruses.

### 4.6. Henipavirus Infection in Pigs

#### 4.6.1. NiV Infection in Pigs

Considering their role in the first outbreak of NiV in Malaysia, pigs have been extensively studied with the aim of understanding virus infection, pathogenesis, and transmission. In natural infection in pigs, NiV is likely transmitted by the oral–nasal route with very high transmissibility observed in pig farms. Infection in pigs is often asymptomatic or mildly symptomatic but occasionally pigs display respiratory symptoms characterized by a severe cough, also referred to as BPS. A more severe febrile respiratory illness with is seen mainly in younger pigs. In some cases, older pigs may display neurological signs including depression, agitation, abnormal posture, muscle spasms, or seizures [311]. Mortality rates vary between 1% and 40% depending on the age of the animal [150].

Experimental studies of NiV infection in pigs via oral or ocular pathways appear to reflect natural infection, with most infected animals remaining asymptomatic, despite viral shedding form the nose and throat 3–7 days post-infection [146]. Subcutaneous infection is linked to increased severity and frequency of symptoms [17]. In pigs infected with NiV, the primary site of viral replication is the respiratory system although it is clear that the virus can spread through the body, especially to the CNS. Bronchial interstitial pneumonia, systemic vasculitis, and localized necrosis of the spleen and lymph nodes have also been observed in pigs with severe clinical disease. Viral antigens have been detected in endothelial cells and smooth muscle cells of the brain, lungs and lymphatic system. Neurons, glial cells and epithelial cells of the upper and lower respiratory tract were also found immunohistochemically positive [146]. Indeed, studies appear to suggest that viral infection begins in nasal tissue and the tonsils before spreading to nearby lymph nodes, the lungs, spleen and the CNS. In keeping with these observations, NiV can be easily reisolated from the nasal and tonsil tissue of infected pigs, but is rarely recovered in the urine or feces [146,276,312]. Viral antigen can be detected in the olfactory nerve 3 days post-infection. Overall, lesions in pigs have been found to be similar to those reported for both NiV and HeV infection in humans and horses.

One interesting difference, however, between NiV infection in swine compared to other species involves the apparent ability of the virus to productively infect porcine lymphocytes. Indeed, necrosis and depletion observed in infected lymphoid tissues in pigs would indicate that the virus can replicate in lymphoid cells. This observation was subsequently confirmed by in vitro infection of porcine peripheral blood mononuclear cells (PBMCs) [275]. This is in direct comparison with data from humans and hamsters in which PBMCs appear to only transport NiV with no viral replication occurring within these cells [109]. In agreement, piglets that succumbed to NiV infection were shown to display a significant drop in CD4+CD8+ T helper cell frequency compared to those that survived. As these lymphocytes are involved in the development of humoral responses, it has been suggested that in surviving animals, a rapid production of virus-specific antibodies might account for a protective response. Such observations would appear to also be in line with those seen for experimental infection of piglets in which lymphoid cell depletion in lymph nodes following inoculation to linked to subsequent secondary bacterial infection in immunodepressed animals [274]. Thus far, however, such a tropism for lymphocytes remains unique to infection in pigs and should be considered when addressing differences in NiV pathogenesis mechanisms in this model.

Of note, a recent study on NiV infection in pigs has suggested important differences in disease progression and severity for infection with NiV Bangladesh compared to NiV Malaysia [313]. This is in contrast to the ferret model, in which both NiV Malaysia and NiV Bangladesh have shown similar pathogenicity. Notable differences include cellular tropism, as evidenced by an absence of viral RNA in the spleen of pigs infected with NiV Bangladesh (in contrast, viral RNA has been detected in the spleens of experimentally NiV Bangladesh-infected hamsters, non-human primates and ferrets), and potentially differing routes of infection of the CNS. The study in question also suggests that in swine NiV-B, invasion of the brain occurs via through viremia with infected monocytes or lymphocytes spreading infection through the lymphatic system [313]. This observation would appear to concur with NiV infection routes of the CNS via the blood–brain barrier in other species [109,146]. Overall, despite an absence of clinical signs over the course of infection, infectious virus was isolated from nasal washes, suggesting that asymptomatic NiV-B-infected pigs would still allow transmission between animals.

#### 4.6.2. HeV Infection in Pigs

Although serological studies have found no evidence of naturally occurring HeV or NiV infections in Australian pigs [270], more recent studies have found evidence of henipavirus exposure in livestock, including in pigs, in Bangladesh [16] and also in both pigs and humans on the African continent [271,314,315,316]. 

The condition caused by both NiV or HeV in pigs appears to be less serious than the condition caused by both viruses in horses but in a single study involving experimental HeV infection in pigs, clinical symptoms were more severe than those generally reported with NiV infection [272]. In pigs, HeV infection via nasal and oral inoculation induces fever, depression, and respiratory symptoms in both 5-week-old farm pigs and in a Göttingen minipig model. In mini pigs, some neurological signs including coordination disorders were also temporarily observed. Lesions ranged from mild to severe and following necropsy, pulmonary congestion and petechiae in multiple organs were found, as well as the presence of syncytia in the respiratory and bronchiolic epithelium. Viral antigen was present in the respiratory epithelial cells, endothelial cells, lymphocytes, macrophages and dendritic cells from submandibular lymph nodes as well as in bronchial and bronchiolar epithelia. Viral RNA was detected by RT-qPCR in nasal, oral, rectal and eye swabs [272]. The virus has been successfully isolated from the tonsils, bronchoalveolar lavage fluid (BALF), lungs and olfactory bulbs, nasal turbinates, submandibular and bronchial lymph nodes and cotton swabs. Pigs expressed neutralizing antibodies 5 to 7 days after infection. The lack of viral RNA in organs that are not directly related to the respiratory system and its draining lymph nodes suggests, however, an absence of systemic spread of infection in these animals.

##### Henipavirus Immune Responses in Pigs

Our understanding of the host immune response to henipaviruses in swine is largely based on vaccination studies [273,276,277]. Both the humoral immune response and development of neutralizing antibodies and cellular responses have been suggested to be vital components of a protective immune response in an infected swine host [274,275,276]. It is clear that pigs are able mount an effective immune response in both natural and experimental settings [14,317]. As mentioned above, and in contrast with many other studied species, in pigs, it appears that NiV can infect a range of porcine immune cells, including monocytes, macrophages, NK cells and T cells. In the same study, NiV-infected primary lymphocytes also failed to induce IFN-alpha [275]. Infection of immune cells by NiV is likely to have a negative impact on the development of adaptive immune responses. Indeed, in pigs, the development of antibodies is associated with protection against NiV [276] and also with clearance of the virus and recovery. In swine, detectable neutralizing antibodies appear around 7 to 10 days post-infection and experimentally infected pigs develop high antibody titers by day 16 dpi. However, despite the presence of neutralizing antibodies, studies have shown that NiV can still be isolated from animal sera 24 days after inoculation and that viral RNA can still be detected 29 days following infection [274].

### 4.7. Experimental Henipavirus Infection in Mice

Standard laboratory mouse models (C57BL/6, Balb/c) are largely unsusceptible to henipavirus infection with either intranasal or intraperitoneal inoculation, although direct intracranial infection with NiV has been shown to cause fatal infection [23,52]. Differences in resistance to infection, however, have been observed depending on the age of the animals used; both young (10 weeks) and aged mice (>12 months) appear more prone to infection and more likely to display disease symptoms [281]. Indeed, aged mice infected intranasally have been shown to develop antibodies against the virus in the absence of symptoms and virus could be detected in the lungs of animals 2–15 days p.i. [282]. Likewise, young mice can develop viral encephalitis after intranasal inoculation with high doses of HeV. As mice have been shown to express functional henipavirus ephrin B2 and B3, it appears that such the lack of susceptibility to clinical disease is occurring at a post-entry step. Indeed, some important observations involving henipavirus pathogenesis and immune responses against this virus have come from infection of IFNAR-KO mice lacking the type I interferon-alpha receptor subunit [23]. In this model, mice were shown to develop clinical symptoms and pathology including weight loss, behavioral differences such as agitation or depression, and neurological signs (aggression, locomotor difficulties or paralysis) [23]. Mice developed vasculitis, meningitis, and bronchial interstitial pneumonia. Virus can be found in numerous organs including the brain, lungs, spleen, and liver with the highest titers found in the brain and lungs. 

#### Henipavirus Immune Responses in Mice

With HeV, seroconversion was observed in surviving IFNAR-KO mice 3 weeks p.i. with both intraperitoneal and intranasal inoculation. Higher Ab titers were seen in 4-week-old mice compared to older animals. Mice that succumbed to infection within the first week of infection did not generate a neutralizing antibody response [23]. Going further, a recent study has again highlighted the important role of type-I IFN in innate defense against henipaviruses in this model [283]. This study suggests a key role of both the mitochondrial antiviral protein signaling (MAV) pathway and of myeloid differentiation primary response 88 (MyD88) adaptors in IFN production and in the control of NiV replication in mice.

Despite a lower susceptibility to infection than some other animal models, mice have been widely used in henipavirus drug treatment and vaccination studies, including for an mRNA vaccine candidate, as well as in studies of viral infection and spread [121,278,279,280,284,285,286,287]. Results with both henipavirus VLPs and soluble henipavirus glycoproteins have shown that mice are able to develop both neutralizing antibodies and CD4 and CD8 T cell responses to viral antigens [279,287]. 

### 4.8. Experimental Henipavirus Infection in Hamsters

Due to their sensitivity to both NiV and HeV infection and their ability to reproduce the pathology associated with infection in humans, golden (Syrian) hamsters have widely been used to study the etiology of henipavirus disease [93,291,293,295,308,318]. When infected with NiV or HeV, hamsters show clinical signs of infection in both the respiratory system (dyspnea, nasal discharge) and the nervous system (paralysis, tremors, convulsions). Both intranasal and intraperitoneal inoculation of hamsters result in the development of fatal neurological symptoms although disease progression depends on the exact dose and route of inoculation [22,291]. Together with thrombosis, vasculitis, and evidence of viral replication via syncytia in the blood vessels of multiple organs, severe pathological lesions can be found in the brain of fatally infected animals. At late-stage infection, virus and/or viral RNA can be recovered from most organs and urine, but viremia is not usually detected [291]. In the case of HeV infection, age seems to affect hamster susceptibility; 11-week-old hamsters have a prolonged course of illness compared to 7-week-old hamsters and require 10-fold higher doses of HeV to achieve complete mortality [289,293,319].

The cytopathogenicity of NiV in this animal model appears to be primarily influenced by the distribution of its cellular receptors, as viral antigen could be detected in small and medium-sized arteries but not in the veins of Syrian hamsters [111]. Some evidence from the hamster model suggests that viral transport may occur via infection of sensory neurons with animals receiving intranasal inoculation [147]. Likewise, hamsters infected with NiV through the consumption of contaminated, artificial palm sap showed evidence that the virus could invade the CNS via sensory neurons [320]. 

The hamster model has also helped to provide important information concerning the pathologies created by henipavirus infection but also concerning the host immune response to infection. Indeed, the hamster model was one of the first in which such responses were studied in detail [291]. Studies have brought to light a general increase in inflammatory signaling and immune cells in the lungs and brain as well as in other organs of infected animals [120]. Other studies have used hamsters to address the role of NiV nonstructural proteins in infection [124]. 

Further studies have compared pathogenicity of NiV Malaysia and NiV Bangladesh strains [111,292]. Whilst one study suggested that with intraperitoneal injection, NiV Malaysia displays faster replication and thus increased pathogenicity in the hamster model, despite very similar clinical signs for the two viral strains, a second study has observed similar pathologies using oronasal inoculation.

#### Henipavirus Immune Responses in Hamsters

The importance of having functional IFN during NiV infection has been shown in hamsters where previous stimulation with an RNA analogue protected them against lethal NiV [296].

A recent study confirmed the ability of NiV to suppress expression of innate immunity-related interferon response genes in endothelial cells, including STAT 1 and 2, CXCL10, interferon-α 7 (Ifna7) or interferon inducible GTPase 1 [140]. It has also been shown that host immune response genes, including IL-4, CXCL10, IL-6, TNFα and IFNγ, are more active with NiV Malaysia infection compared to NiV Bangladesh [292].

As well as in infection and transmission studies, the hamster model has also been used extensively to evaluate therapeutic approaches including serotherapy and vaccination [288,289,293,296,297,298,299,300,301,302,303,319,321]. The majority of these studies show that single doses of henipavirus vaccines, administered even at late stages prior to virus challenge, can achieve protective immunity and that serum from protected animals can further be used to provide passive protection against infection. Such results would suggest a key role for adaptive immune responses in preventing fatal henipavirus infection in animals.

### 4.9. Experimental Henipavirus Infection in Guinea Pigs

Although guinea pigs were one of the first animal models used to study HeV infection, clinical signs of infection seen in this small animal model are significantly different from the effects seen in cases of human or horse infection with the same pathogen. In guinea pigs, HeV caused a systemic vascular disease with little or no evidence of respiratory edema [21,52,237]. Histologically, the disease touches the arteries and veins as well as many other organs including the kidneys, spleen, bladder and gastrointestinal tract but also the lymph nodes and muscle tissue. Not all animals develop clinical signs or die with infection [52]. Both symptoms and mortality increase upon subcutaneous inoculation routes whereas intracutaneous inoculation does not result in infection, although a majority of animals seroconvert [21].

Similar results are seen with NiV infection of guinea pigs, for which nasal inoculation fails also to produce clinical signs. Intraperitoneal inoculation is considered more effective with either symptoms of weight loss and a temporary fever followed by recovery or continued weight loss and death. Following infection NiV can be isolated from numerous organs and blood [159]. 

#### Henipavirus Immune Responses in Guinea Pigs

Not much information exists regarding the immune responses during henipaviruses infection in guinea pigs. Extensive inflammation has been described to be present in the urogenital system of NiV infected guinea pig [304]. Another study showed that surviving animals develop neutralizing antibody titers against the virus [159]. Of note, guinea pigs have also been used to study infection with the related CedV. Although serum-neutralizing antibodies were found 21 days p.i. (2/4 animals), animals did not display any clinical symptoms of infection [308].

A summary of the innate and adaptive immune responses described for henipavirus infection for the major host species and animal models can be found in Table 2.

### 4.10. Conclusions

In order to understand the underlying mechanisms of henipavirus pathogenesis and recrudescence, experimental animal models are essential. Despite the broad tropism displayed by henipaviruses, not all animals are affected equally. Thus, the multiple existing animal models show different suitability for modeling different features of henipavirus infection and disease, including shedding, pathogenesis, clinical disease, and transmission or the efficacy of antiviral treatments or vaccines.

For example, while cats are a good model to study the respiratory disease observed during henipavirus infection, guinea pigs better recapitulate the neurologic disease seen with natural infection. In contrast, AGM, ferret, and hamster models closely mimic both respiratory and the neurological disease progression. With regards to countermeasure development, since to date no lethal mice model that fully recapitulates henipavirus disease features exists, the main models used are hamsters, ferrets and ultimately the AGM model. Therefore, animal models for HeV and NiV studies should be used according to the purpose of the study. In the future, further development of species-specific immunological tools will surely contribute to an increase and broader use of animal models to study different aspects of henipavirus infections. Importantly, while to date a few experimental studies have been performed with bats, we would like to emphasize here the importance in pursuing research studies where the immune differences between bats and other animals, including humans, affected by henipaviruses are addressed. The availability of bat-specific immune tools would be a considerable contribution to this field. An increased understanding of how some animal species, especially bats, are able to successfully control infection with highly pathogenic viruses such as the henipaviruses, will also provide new insights into how to prevent or treat infection in humans and livestock.

## Figures and Tables

**Table 1:** Animal models of HeV and NiV infection.

Animals	Type of Infection	Onset of Illness (Days)	First Clinical Signs	Symptoms	Virus-Positive Tissues or Fluids	References
**Bat** **(*P. alecto, P. poliocephalus*)**	s.c.	N/A	vascular lesions	N/A	blood, kidney, urine, uterus	[67,68,149]
	s.c.	N/A	mild nephritis, focal vasculitis, cell infiltration in liver	N/A	kidney, urine, rectum	[42,159]
**Cat**	o.i., i.n., s.c.	5–7/9	inappetence, increased respiratory rate	severe respiratory disease, pneumonia, hemorrhagic lungs, vascular lesions, syncytial cells	arteries, veins, lung, spleen, kidney, brain, gastrointestinal tract, urine	[52,149,236,237]
	o.n.i., s.c.	4–10/12	fever, respiratory and neurological disease, depression, constipation,	ulcera, inflammation, meningitis, meningeal vasculitis	oropharynx, tonsil, trachea, lung, brain, kidney, liver, uterus	[17,238,239,240]
**Dog**	s.c. natural	N/A	no signs of ill health, although wincing several times	reddening and dark patchy discoloration in the lung and the tonsils, white streaks in kidney, spleen and liver inflammation, fibrinoid necrosis, vasculitis and inflammatory infiltrates in kidney, brain, LN, spleen, liver, intestine, lung, expanded meninges	kidney, brain, lymph nodes, spleen, and liver Equivocal NAb	[52,241,242]
	natural	N/A	fever, respiratory distress, conjunctivitis, nasal discharge	severe pulmonary edema, atrophy, vascular degeneration, syncytia, necrosis, glomerulonephritis, meningitis	N/A	[46,240,243,244]
**Ferret**	o.n.i., i.n.	6–9	fever, depression, serous nasal discharge, dyspnea, hind limb paresis and generalized tremors	cutaneous petechiation, s.c. edema in head and neck, pulmonary petechiation, hemorrhagic lymph nodes, systemic vasculitis, necrosis, splenitis, bronchoalveolitis, endothelial and epithelial syncytial cells	kidney, heart, bladder, LN, lung, spleen, brain, nose, endothelial cells, neurons, bronchoalveolar epithelium, urine	[190,245,246,247]
	o.n.i., i.n.	5–9	pyrexia, depression, cough, dyspnea, hind limb paresis, generalized tremors	multisystemic inflammatory lesions in respiratory tract, spleen, kidneys and liver, neurologic disease, meningitis, encephalitis, hemorrhaging, necrosis, syncytia, bronchoalveolitis, tonsillitis, nasopharyngitis, thrombocytopenia, multisystemic vasculitis	nasal turbinates, pharynx, retropharyngeal lymph nodes, spleen, lung, liver, kidney, LN, uterus, ovaries, heart, brain, bladder, mouth, rectum, vascular endothelium, feces, neurons, glial cells, urine	[246,247,248,249,250,251,252,253]
**Horse**	s.c., i.n., o.n.i., i.v.	5–16	fever, high cardiac rates, depression, dyspnea, recumbency, loss of appetite, neurological signs	lung edema, thrombosis, hemorrhage, tissue necrosis, syncytia, vascular damage	kidney, lung, mouth, nasal cavities, urine	[48,49,89,149,254,255,256]
	natural	N/A	neurological signs	vascular damage, meningitis (only brain and spinal cord were analyzed)	brain, spinal cord seroconversion	[240,257]
**Monkey (African green monkey, AGM)**	i.n., i.t.	7	piloerection, respiratory distress, nasal discharge, depression, seizures, muscle fasciculations	severe systemic vasculitis, necrosis, hemorrhage and edema in most organs, splenomegaly, hemorrhaging, syncytial cells, meningitis	tonsils, trachea, lungs, heart, liver, spleen, kidney, pancreas, intestine, LN, brain, testes/ovaries, bone marrow, urine	[115,258,259]
	i.n., i.t., o.i.	7–12	fever, loss of appetite, respiratory disease, lethargy, rash, depression, behavioral changes	severe systemic vasculitis, hemorrhage and edema in most organs, thrombocytopenia, meningitis	blood, trachea, lungs, heart, liver, spleen, kidney, pancreas, intestine, LN, brain, testes/ovaries, bone marrow, rectum, urine	[24,59,259,260,261,262,263,264,265,266,267,268]
**Monkey (Squirrel Monkey)**	N/A	N/A	N/A	N/A	N/A	N/A
	i.v., i.n.	7–19	respiratory disease, loss of appetite, depression, uncoordinated motor movements	inflammation of lung parenchyma, mild vasculitis	spleen, liver, lung, heart, bladder, kidney, LN, spinal cord, brain	[269]
**Pig**	i.n., o.n.i.	4/5	fever, loss of appetite, cough, respiratory distress, depression, uncoordinated movements	pulmonary edema, hemorrhages in lung, kidney and LN, syncytial cells, inflammation, vasculitis, necrosis	Tonsils, lung, nasal turbinates, LN, olfactory bulb, mouth, ocular secretions, rectum	[270,271,272,273]
	o.n.i., o.i., s.c., i.n., oc.i.	7–21	fever, nasal discharge, coughing, locomotor disturbances, agitation, muscle fasciculations, paresis, seizures	systemic vasculitis, vasculopathy, alveolitis, thrombosis, cell necrosis, pulmonary edema and inflammation, renal tubular degeneration, syncytia, meningitis,	tonsils, lung, LN, olfactory bulb, nose, oropharynx, spleen, endothelium, lymphatic vessels, kidneys, brain, blood, urine	[14,17,146,244,273,274,275,276,277,278]
**ID mice (IFNAR-KO, NSG)**	i.n., i.p.	3–21	agitation, lack of grooming, grimace, loss in body weight, lordosis, aggression, locomotor disability, head tilt, and paralysis	lung and brain congestion, hemorrhages, vasculitis, necrosis, meningitis, encephalitis	brain, lung, spleen, liver	[23]
	i.p., i.n., i.cer.	6–10	agitation, lack of grooming, grimace, loss in body weight, lordosis, aggression, locomotor disability, head tilt, and paralysis	inflammation, edema, focal necrosis in lung and vasculitis microscopic lesions in the brain, liver and kidney inflammation, syncytial cells, meningeal inflammation	brain, lung, spleen, liver	[23,121,279,280]
**IC mice (C57BL/6, BALB/c, CB6F1/J)**	i.n., s.c.	10–21	depression, ataxia, hypersensitivity, and tremors	necrosis, ulceration, encephalitis	respiratory tract, olfactory epithelium, brain	[52,281]
	i.n., i.p. i.cer.	4	N/A	subclinical, self-limiting respiratory infection	lung, spleen	[23,278,282,283,284,285,286,287]
**Hamster**	i.p.	3–25	breathing difficulties, paralysis, and trembling limbs	pulmonary edema, inflammation in lung and spleen, necrosis, syncytia cells, meningitis	lung, heart, liver, spleen, brain, serum, urine	[288,289,290,291]
	i.n., i.p., a.e., fom., d.c.	4–15	breathing difficulties, imbalance, limb paralysis, lethargy, muscle twitching	damage in lung, liver, kidney, heart, and brain, fibrinoid necrosis with surrounding inflammation in blood vessels, syncytial cells, necrosis, vasculitis, thrombosis, meningitis	lung, kidney, spleen, liver, heart, spinal cord, brain, urine	[22,93,111,124,147,288,289,292,293,294,295,296,297,298,299,300,301,302,303]
**Guinea pig**	s.c., i.n., i.d.	7–15 days	inappetence, increased respiratory rate, head tilt, ataxia, torticollis, depression	pneumonia, cyanose, oedema in gastrointestinal tract, systemic vascular disease in arteries, veins, lung, kidney, spleen, lymph nodes, syncytia in lungs	arteries, veins, lung, kidney, spleen, lymph nodes	[21,52,160,237]
	i.p., i.n.	7–9 days	ruffled hair, weight loss, abnormal behavior, ataxia	oedema, systemic vasculitis, endothelial syncytial cells, cell necrosis, lung hemorrhages	heart, spleen, kidney, lung, brain, LN, thymus, blood, ovaries, uterus	[22,159,304]
**Rat**	s.c.	N/A	no histological lesions	N/A	virus not observed Equivocal NAb	[52]
	N/A	N/A	N/A	N/A	N/A	N/A
**Rabbit**	s.c.	N/A	no histological lesions	N/A	virus not observed	[52]
	N/A	N/A	N/A	N/A	N/A	N/A
**Chicken**	s.c.	N/A	no histological lesions	N/A	virus not observed	[52]
	allantoic or yolk sac inoculation	5–7	only reported in embryos: 4–7	only reported in embryos: brain hemorrhage, congestion and hemorrhage in the skin of the toes and in the kidneys, syncytial cells, necrosis	only reported in embryos, lung, heart, liver, kidney, spleen, skin, CNS, blood vessels	[305]

s.i.: subcutaneous injection; i.cer.: intracerebral; i.d.: intradermal; o.c.i.: ocular infection; i.n.: intranasal; i.t.: intratracheal; o.i.: oral inoculation; o.n.i.: oronasal injection; i.p.: intra peritoneal; a.e.: aerosol exposure; fom.: fomites; d.c.: direct contact; Nab: neutralizing antibodies; LN: lymph nodes; CNS: central nervous system; N/A: not analyzed; IC: immunocompetent; ID: immunodeficient.

**Table 2:** Innate and adaptive immune responses during HeV and NiV infection.

Host		Human	Bat	Cat	Dog	Ferret	Horse	Monkey	Pig	IC Mice	Hamster	Guinea Pig
**Innate**	**Inflammation**											
	**Interferon expression**											
	**Complement**											
**Adaptative**	**Lymphocyte activation**											
	**Immunoglobulines**											
	**Neutralizing antibodies**											
	**Lymphopenia**											

IC: immunocompetent.
Inhibition or decreased levels or not present with henipavirus infectionActivation or presence with henipavirus infectionNot yet described with henipavirus infection

## Data Availability

Not applicable.
